# A Case of Ancient Lateral Dislocation Outward of the Radius and Ulna

**Published:** 1858-04

**Authors:** W. H. Mason


ART. IV.—*A Case of Ancient Lateral Dislocation outward of the Radius and Ulna.* Presented to the Class at the Buffalo Medical College, Jan. 28th, 1858, by Prof. FRANK H. HAMILTON. Reported by W. H. MASON, Jr.

The following case is of such rare occurrence that it is deemed worthy of being placed upon record:

Francis Banfield, aged 22 years, a resident of Cuba, Alleghany county, on the 31st of September last, nearly four months since, fell from the sweep of a threshing machine to the ground, a distance of about five feet, striking upon the palm of his hand, his arm being extended in front of him. On rising he found his arm forcibly flexed and abducted. He straightened it without difficulty, and it assumed the position it now occupies. A physician was called and saw the patient an hour and a-half after the accident, who pronounced it a case of dislocation of the radius and ulna, and made efforts at reduction, which he continued from 8½, A. M. until 2, P. M., a period of five and a-half hours, to no purpose, when he abandoned the attempt.

During the attempt at reduction, the extension was made at times with the arm flexed, and at others extended.

At 9, P. M., another physician was called, who made efforts at reduction until 3, A. M., upwards of six hours, at which time he also abandoned the attempt.

On the third day another physician, the patient being under the influence of ether, made efforts at reduction for twenty minutes, when he pronounced it in place, and applied a bandage.

From the patient's account the arm was swollen to such an extent as to render this point difficult to determine

On the fifth day the first physician was called, and believing that he discovered a grating, pronounced it a fracture of the external condyle.

The limb now presents the following appearances: Arm nearly straight; a moderate outward deflection of the forearm upon the arm, existing while the arm is at rest, which may be increased by the surgeon to an angle of 45°. The power of pronation and supination is nearly perfect.

The inner condyle projects an inch to the ulnar side; the head of the radius, completely removed from its socket, projects to an equal extent on the radial side. The top of the olcranon process is an inch higher than the top of the inner condyle, so that the radius and ulna are carried upward as well as inwards.

Prof. Hamilton remarked that in his opinion there was no fracture of the external condyle. As in that case, the arm would be *permanently* deflected outward to a much greater extent. For although this arm may be deflected outward by the surgeon to an angle of 45°, still the degree of mobility which exists would be adverse to the supposition of its being a fracture of the external condyle. The condyles also can be plainly felt in their natural situations, which would not be the case, if a fracture of the external condyle existed. Prof. Hamilton advised that no farther attempts should be made at reduction.

